# Getting out of the 1950s: rethinking old priorities for staffing in critical care

**DOI:** 10.1186/s13054-020-03426-z

**Published:** 2020-12-14

**Authors:** Hannah Wunsch

**Affiliations:** 1 Department of Critical Care Medicine, Sunnybrook Health Sciences Centre, 2075 Bayview Ave, Rm D108, Toronto, ON, ON M5R 3B2 Canada; 2 Department of Anesthesia and Interdepartmental Division of Critical Care Medicine, University of Toronto, Toronto, ON Canada; 3 Sunnybrook Research Institute, Toronto, ON Canada; 4 Department of Anesthesiology, Columbia University, New York, NY USA

Medicine as we practice it is shaped by routines from decades ago; the attending physician on morning rounds, trailed by nurses, residents, and medical students. In those earlier days, there was little that could be done for someone who was struggling to breathe except perhaps get a chest x-ray and give them oxygen. Heart attacks in the 1950s were treated with bedrest [1], and a description by an intern in 1951 describes routinely finding cardiology patients dead in the morning when coming to draw blood [2].

The physician “on service” in the intensive care unit (ICU) well into the 1990s (and into the new millennium in some places) was often attending in the unit for a month at a stretch. Once rounding finished, the attending would usually leave, to reappear again the next morning. At least in many US academic centers, attendings were never or rarely called for deaths, deteriorations, or difficult families, and certainly not at night. The continuity of daily rounds by the same physician was prized above all else. A culture developed that hand-offs spelled death, and that continuity of care was to be maintained at all cost.

But the modern critically ill patient may have blood drawn every 4–6 h or even more frequently, continuous monitoring, 20 different medications, as well as mechanical ventilation, dialysis, and possibly extracorporeal life support. Families expect to be updated for all critical events; consent must be obtained for every procedure. With the ability to provide endless unnatural prolongation of life, family meetings about preferences for care are essential and complex. Deaths, deteriorations and families with conflict all (often) require involvement of an experienced physician.

To deal with these demands, we have seen a gradual change in the approach to staffing by intensivists. First, the month became 2 weeks and then 1 week. In a recent survey of 23 North American critical care organizations, the range of consecutive shifts allowed was 1–14, with a median of 7, suggesting that the “norm” is now a week at a time [3]. In a survey of Australian ICUs (*n* = 109), 43 (39.4%) had intensivists scheduled to work for 7 or more consecutive days, with a median of 5 days [4]. The specialty has evolved to meet these challenges of twenty-first-century critical care, but the optimal model is very unclear.

There will always be large benefits of continuity, if for no other reason than the reassurance it provides to patients and families to feel known in a foreign environment; the importance of this aspect of care cannot be over-emphasized and also provides satisfaction to clinicians. But continuity with such complex patients also presents challenges. Patients are so complicated that issues can be missed; deteriorations may go unnoticed, or the realities of a trajectory may be denied. These issues are not due to poor care, but because of the onslaught of information and speed of modern medicine, and the realities of fatigue. In a study of cross-coverage among fellows in a single center, outcomes were better for patients who had some care by a cross-covering physician at night versus those who did not [5]. If one believes in the continuity mantra, such a signal should not exist. A cluster-randomized trial demonstrated no difference in ICU length of stay or mortality when intensivists worked 2 weeks straight when compared to a model with weekends covered by someone else; moreover, physicians under the 2-week continuous model reported significantly higher burnout [6]. Similarly, a recent survey by Mikkelsen et al. found that burnout was more commonly associated with 14-day rotations than 7-day rotations [7]. Finally, in a recent observational study of Australian ICUs, there was a signal for a shorter ICU length of stay for patients cared for in units that had intensivists working fewer consecutive days, without increases in ICU readmissions or hospital mortality [4]. Such findings may be uncomfortable to those wedded to care continuity, and they are merely hypothesis generating. But most importantly, they suggest we need to more carefully re-examine what matters most for best care when “bed rest for 6 weeks” is no longer our treatment for a myocardial infarction or pneumonia. These studies chip away at the armor of invincibility that is the mantra of continuity.

Our jobs are just not the same as 50 years ago, or even 20 years ago. We must continue to shift and evolve to meet these new demands while accepting that physicians cannot be all things to everyone at all times. Continuity, availability and the number of patients an individual can care for at once are aspects of providing good medical care that will always be in tension. What are the solutions? First is an openness to new considering new models and a willingness to acknowledge that critical care has changed and optimal models for care that balance priorities have therefore also changed [8]. Second is more data. Understanding the true trade-offs and costs associated with different staffing models is essential. While such studies are messy and challenging to interpret, we can only make informed choices when we understand what is gained and what is lost (Fig. 1). Third is recognizing that delivery of critical care has become a team sport; so many other individuals with clinical expertise are essential to the effective care of critically ill patients, that we need to appreciate the roles of all of these individuals. And fourth is to acknowledge that burnout is a real concern. Sacrificing our workforce for a perceived “best” model that does not account for burnout may be a short-term solution but will not be sustainable as we move through COVID-19 and beyond.

**Fig. 1:**
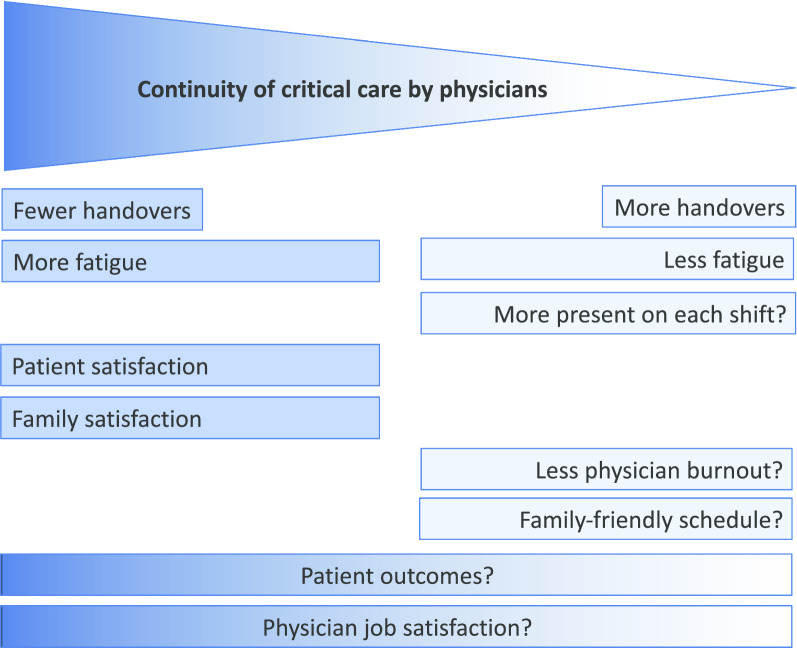
Known and potential trade-offs of different degrees of continuity of care provided by physicians caring for critically ill patients

## Data Availability

Not applicable.

## References

[1] Levine SA (1951). The myth of strict bed rest in the treatment of heart disease. Am Heart J.

[2] Braunwald E (2012). The treatment of acute myocardial infarction: the past, the present, and the future. Eur Heart J Acute Cardiovasc Care.

[3] Lilly CM, Oropello JM, Pastores SM, Coopersmith CM, Khan RA, Sessler CN, Christman JW (2020). Academic leaders in critical care medicine task force of the society of critical care M: Workforce, workload, and burnout in critical care organizations: survey results and research agenda. Crit Care Med.

[4] Gershengorn HB, Pilcher DV, Litton E, Anstey M, Garland A, Wunsch H (2020). Association between consecutive days worked by intensivists and outcomes for critically ill patients. Crit Care Med.

[5] Kajdacsy-Balla Amaral AC, Barros BS, Barros CC, Innes C, Pinto R, Rubenfeld GD. Nighttime cross-coverage is associated with decreased intensive care unit mortality. A single-center study. *Am J Respir Crit Care Med.* 2014;189(11):1395–401. 10.1164/rccm.201312-2181OC 24779652

[6] Ali NA, Hammersley J, Hoffmann SP, O'Brien JM, Phillips GS, Rashkin M, Warren E, Garland A (2011). Midwest Critical Care C: Continuity of care in intensive care units: a cluster-randomized trial of intensivist staffing. Am J Respir Crit Care Med.

[7] Mikkelsen ME, Anderson BJ, Bellini L, Schweickert WD, Fuchs BD, Kerlin MP (2019). Burnout, and fulfillment, in the profession of critical care medicine. Am J Respir Crit Care Med.

[8] Courtright KR, Kerlin MP (2014). Intensive care unit staffing and quality of care: challenges in times of an intensivist shortage. Rev Bras Ter Intensiva.

